# Stress fields around two pores in an elastic body: exact quadrature domain solutions

**DOI:** 10.1098/rspa.2015.0240

**Published:** 2015-08-08

**Authors:** Darren Crowdy

**Affiliations:** Department of Mathematics, Imperial College London, 180 Queen's Gate, London SW7 2AZ, UK

**Keywords:** plane elasticity, pores, stress fields, quadrature domains

## Abstract

Analytical solutions are given for the stress fields, in both compression and far-field shear, in a two-dimensional elastic body containing two interacting non-circular pores. The two complex potentials governing the solutions are found by using a conformal mapping from a pre-image annulus with those potentials expressed in terms of the Schottky–Klein prime function for the annulus. Solutions for a three-parameter family of elastic bodies with two equal symmetric pores are presented and the compressibility of a special family of pore pairs is studied in detail. The methodology extends to two unequal pores. The importance for boundary value problems of plane elasticity of a special class of planar domains known as quadrature domains is also elucidated. This observation provides the route to generalization of the mathematical approach here to finding analytical solutions for the stress fields in bodies containing any finite number of pores.

## 1. Introduction

There is a rich tradition in the field of linear plane elasticity of making use of analytic function theory, coupled with conformal mapping techniques, to find the stress distributions in two-dimensional elastic media. The monographs by Muskhelishvili [1] and Sokolnikoff [2] provide detailed accounts of the scope of such mathematical methods in these fields. A more recent treatment was given by England [3].

One application of this methodology is to the study of the stress distribution around pores or voids [4], a problem of particular significance for rock mechanics [5], ceramics [6] and bones [7]. It is a problem of classical interest [8]. An extensive study of single two-dimensional pores of various shapes was performed by Zimmerman [9].

Zimmerman used conformal mapping to find the two complex potentials determining the stress distribution in the elastic medium around an isolated pore with special interest in the so-called pore compressibility, that is, the change of the hole area under a hydrostatic stress. He focused there on the case of hypotrochoidal holes. In more recent extensions of that work, Ekneligoda & Zimmerman have studied the compressibility [10] and the shear compliance [11] of a generalized family of isolated non-circular pores with *n*-fold rotational symmetry including those exhibiting high curvature, or near-cuspidal, boundary regions. The idea of the latter work is to include more terms in a series expansion of a conformal mapping function from a unit disc pre-image domain. In view of the highly irregular geometrical structure of pores as viewed from scanning electron micrographic images, there is great interest in determining the stress distribution around such *non-circular* pores. Other contributions in this vein have been made by Jasiuk *et al.* [12] and Kachanov *et al.* [13]. A prevailing feature in much of this work is the consideration of special classes of elasticity domains whose shapes are encoded in conformal mappings having a functional form conducive to finding analytical solutions of the boundary value problem for the associated stress field potentials.

Similar problems for the stress fields around multiple interacting pores in an unbounded elastic medium have also been studied, especially in the two-pore case, but the literature is much more limited. Analytical solutions are known for the case of two pores: Ling [14] found the stress distribution around two equal circular pores; Haddon [15] generalized to the case of unequal circular pores (see [16]). A useful review of the literature associated with the two-pore problem has been given by Panasyuk & Savruk [17].

**Figure 1.**
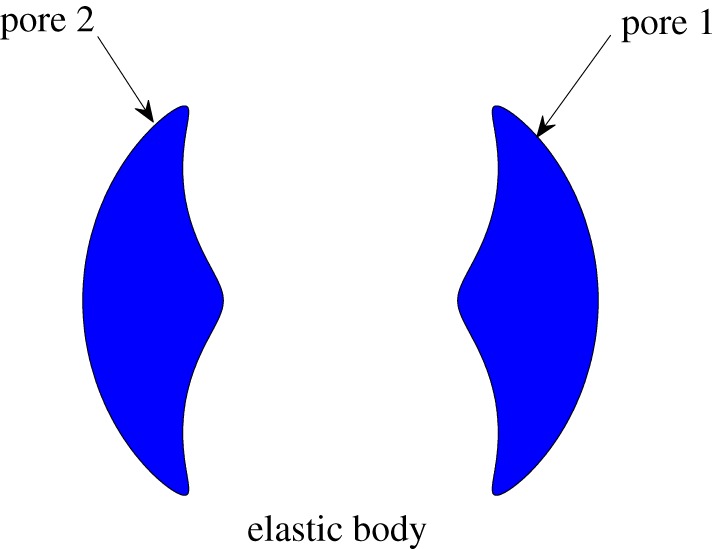
The geometry under consideration: a two-dimensional infinite elastic body with two symmetric, equal-area, non-circular pores.

This paper shows how to derive analytical solutions for the stress distribution around two *non-circular* pores; the geometry is depicted in figure 1. We produce a three-parameter family of non-circular, but equal-sized, pores in an unbounded elastic medium where the complex potentials determining the stress distribution can be written down explicitly in terms of a special function known as the *Schottky–Klein prime function* associated with a pre-image annulus.

While we focus here on a specific class of shapes, our approach is very general and is extendible to much wider families of shapes including two pores of unequal sizes.

More generally, the doubly connected region exterior to the family of pore pairs considered here has mathematical significance: they are examples of doubly connected *quadrature domains*. Quadrature domains have been studied for their abstract mathematical properties [18], but they have also been found to have application in a diverse array of physical problems such as the so-called Hele–Shaw problem of fluid mechanics [19]. Indeed, the author has surveyed the many other areas of fluid dynamics, beyond the Hele–Shaw problem, where quadrature domains play a role [20]. The present work appears to be the first to point out, and showcase, the role of quadrature domains in problems of plane elasticity. Importantly, our methodology also extends to finding the stress distributions around *any finite number* of pores in an unbounded medium. This matter is discussed in more detail in §7.

## 2. Function theory in an annulus

To solve the linear elasticity problems of interest here, we need to introduce some convenient functions in the annulus *ρ*<|*ζ*|<1. All those needed can be constructed from the basic function

2.1 $$P(\zeta )\equiv (1-\zeta )\hat{P}(\zeta ),\;\hat{P}(\zeta )\equiv \prod_{k=1}^{\infty }(1-\rho^{2k}\zeta )(1-\rho^{2k}\zeta^{-1}).$$

Standard techniques [21] can be used to confirm that this infinite product is convergent for all finite *ζ*≠0 and 0<*ρ*<1. It is easy to verify directly from the definition (2.1)—see appendix A for details—that

2.2 $$P(\rho^{2}\zeta )=-\zeta^{-1}P(\zeta ),\;P(\zeta^{-1})=-\zeta^{-1}P(\zeta ).$$

An important feature of the function *P*(*ζ*) is that it vanishes when *ζ*=1, and also at all points *ζ*=*ρ*^2*n*^, where *n* is any non-zero integer.

We also introduce

2.3 $$K(\zeta )\equiv \frac{\zeta P^{\prime }(\zeta )}{P(\zeta )},$$

which is the logarithmic derivative of *P*(*ζ*) multiplied by *ζ*, and the prime notation denotes differentiation with respect to *ζ*. It has a simple pole at *ζ*=1 with residue +1, that is, near *ζ*=1,

2.4 $$K(\zeta )=\frac{1}{\left(\zeta -1\right)}+\text{locally analytic function}.$$

From (2.2), it follows that

2.5 $$K(\rho^{2}\zeta )=K(\zeta )-1,\;K(\zeta^{-1})=1-K(\zeta ).$$

Finally, it is also useful to consider a second derivative of *P*(*ζ*) in the form of

2.6 $$L(\zeta )\equiv \zeta \frac{dK(\zeta )}{d\zeta }.$$

Properties (2.5) can be used to verify that

2.7 $$L(\rho^{2}\zeta )=L(\zeta ),\;L(\zeta^{-1})=L(\zeta ).$$

*L*(*ζ*) has a second-order pole at *ζ*=1 with strength −1, that is, near *ζ*=1,

2.8 $$L(\zeta )=-\frac{1}{{\left(\zeta -1\right)}^{2}}+\text{locally analytic function}.$$

*L*(*ζ*) is an example of a *loxodromic function* [22]: a function *H*(*ζ*) is defined to be a loxodromic function if it is meromorphic everywhere inside (and on the boundary of) some *fundamental annulus* *ρ*≤|*ζ*|<*ρ*^−1^ and satisfies the functional relation

2.9 $$H(\rho^{2}\zeta )=H(\zeta ).$$

The annulus *ρ*≤|*ζ*|<*ρ*^−1^ is called fundamental because, given the singularity structure of *H*(*ζ*) in this annulus, its singularities in all other annuli filling out the complex *ζ*-plane follow from (2.9). For *L*(*ζ*), because its only singularity in the fundamental annulus (or, strictly speaking, on its boundary) is a second-order pole at *ζ*=1, and because it satisfies (2.9), then *L*(*ζ*) qualifies as a loxodromic function.

The function *K*(*ζ*) in (2.3), although not itself loxodromic, will be our main tool later in constructing loxodromic functions having simple pole singularities.

## 3. Conformal mapping from an annulus

Consider the special class of unbounded elastic bodies exterior to two finite area pores described by conformal maps from the annulus *ρ*<|*ζ*|<1 having the form

**Figure 2.**
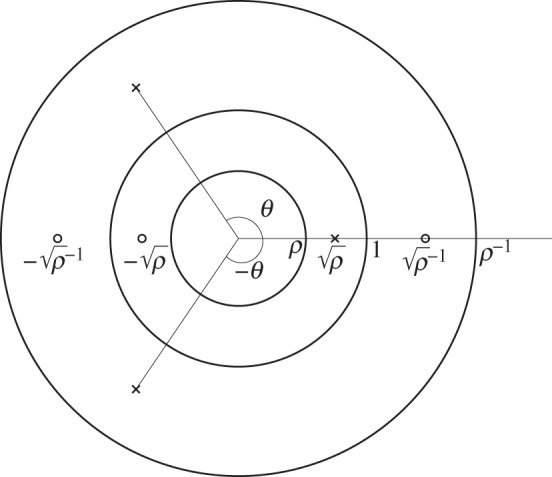
Locations of the three poles (crosses) and three zeros (small circles) for the conformal mapping (3.1). In the annulus *ρ*<|*ζ*|<1, which is the pre-image of the elastic body, the mapping has a pole at $\sqrt{\rho }$ and a zero at $-\sqrt{\rho }$. It also has two simple poles, at ${\sqrt{\rho }}^{-1}\;e^{\pm i\theta }$, in the unphysical annulus 1<|*ζ*|<*ρ*^−1^ as well as two simple zeros at $\pm {\sqrt{\rho }}^{-1}$.

3.1 $$z=z(\zeta )=R\left[\frac{P(-\zeta /\sqrt{\rho })P(-\zeta \sqrt{\rho })P(\zeta \sqrt{\rho })}{P(\zeta /\sqrt{\rho })P(\zeta \sqrt{\rho }\;e^{i\theta })P(\zeta \sqrt{\rho }\;e^{-i\theta })}\right],$$

where *R*,*θ* and *ρ* are real constants. (There is a minor abuse of notation here in that *z* is taken to denote both the complex coordinate in the physical plane *and* the conformal mapping function, but this should not cause confusion.) From its form as a ratio of products of *P* functions, the poles and zeros of the function (3.1) are clear: because *P*(*ζ*) vanishes simply when *ζ*=1 then, restricting to the poles and zeros in the annulus *ρ*<|*ζ*|<1/*ρ*, we see that there are three simple zeros at $-\sqrt{\rho },\pm {\sqrt{\rho }}^{-1}$ and three simple poles at $\sqrt{\rho },{\sqrt{\rho }}^{-1}\;e^{\pm i\theta }$. Figure 2 shows a schematic illustrating the pole and zero locations of (3.1) in the annulus *ρ*<|*ζ*|<*ρ*^−1^.

Under the mapping (3.1), the circle |*ζ*|=*ρ* (called *C*_1_) and the circle |*ζ*|=1 (called *C*_0_) are transplanted to the pore boundaries, the point $\zeta =\sqrt{\rho }$ maps to z=∞ and $\zeta =-\sqrt{\rho }$ maps to the origin *z*=0. Not all choices of parameters are physically admissible though: we must restrict to parameter choices for which the image of the annulus *ρ*<|*ζ*|<1 under the mapping is one-to-one. A necessary (but not sufficient) condition is that *z*′(*ζ*)≠0 for *ρ*<|*ζ*|<1.

For all choices of admissible parameters, it can be verified that the images of the circles |*ζ*|=*ρ*,1 are rotations of each other by angle *π* about the origin. To see this, note that properties (2.1) of *P*(*ζ*) can be used to show that, for all *ζ*≠0,

3.2 z(ρ/ζ)=−z(ζ),

implying that for each point *ζ* on the circle |*ζ*|=1, there is a point on the circle |*ζ*|=*ρ* having the negative image under the mapping.

For any analytic function *h*(*ζ*), its Schwarz conjugate function $\bar{h}(\zeta )$ is defined by

3.3 $$\bar{h}(\zeta )\equiv \bar{h(\bar{\zeta })}.$$

It is easy to check that, owing to the reflectional symmetry of its pole and zero locations about the real *ζ*-axis, the conformal mapping (3.1) satisfies

3.4 $$\bar{z}(\zeta )=z(\zeta ),$$

which is consistent with the reflectional symmetry of the image domain about the real *z*-axis.

Of crucial importance for our development is the observation that, on use of (2.2), it can be checked that (3.1) satisfies the functional relation

3.5 $$z(\rho^{2}\zeta )=z(\zeta )$$

for all choices of the parameters *R*,*θ* and *ρ*. Because (3.1) can also be seen to be meromorphic for all *ζ*≠0 then *z*(*ζ*) is a loxodromic function. It is this feature of the conformal mappings that causes the domains whose images they represent to be called *quadrature domains* (see §7 for more discussion). Given that *z*(*ζ*) satisfies (3.5), it should be clear that, if *z*(*ζ*) is meromorphic, it is enough to study its poles and zeros in the annulus *ρ*<|*ζ*|<*ρ*^−1^ because its poles and zeros in all other annuli will then follow from (3.5). In this way, the poles and zeros depicted in figure 2 are the only ones with which we need be concerned (even though, in fact, the function *z*(*ζ*) has a countable infinity of zeros and poles).

The analysis to follow pertains for *any* choices of the three real parameters *ρ*,*θ* and *R*. However, in the example calculations given later, we focus on the particular choice

3.6 $$\theta =\frac{2\pi }{3}.$$

This restriction reduces the pore shapes studied to a two-parameter family. Even more, we will normalize the domains to be such that the centroid of the two pores are at ±1 on the real axis, thereby further reducing the class of shapes to a one-parameter family. This condition on the centroids fixes *R* as a function of *ρ* and *θ*. Mathematically, *ρ* then serves as the natural governing parameter; altering it corresponds physically to varying the area of the two symmetric pores. As *ρ*→0, the pore areas tend to zero; as *ρ* increases, the area of each grows and the separation between closest points on the pores decreases.

**Figure 3.**
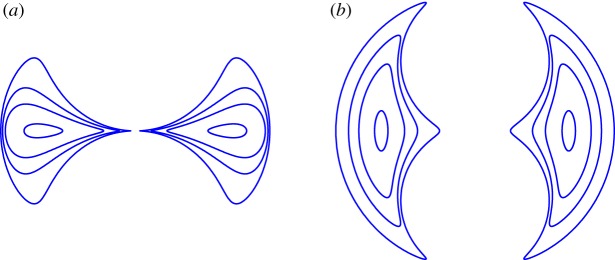
(*a*,*b*) Typical two-pore shapes described by the mapping (3.1) for *θ*=*π*/3 (*a*) and *θ*=2*π*/3 (*b*) and *ρ*=0.005, 0.05, 0.1 and 0.2 (superposed) with centroids fixed. The elastic body is the unbounded doubly connected region exterior to the pores. For small area and *θ*=2*π*/3, the pores are near ellipses with semi-axis ratio 1 : 3.

**Figure 4.**
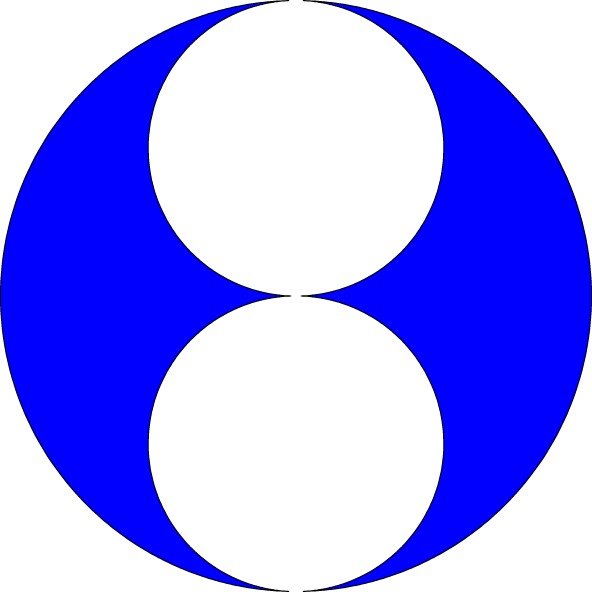
Pore shapes with *ρ*=0.5 and *θ*=2*π*/3. The pores are close to touching at three near cusps. The two ‘outer’ pore boundaries together appear to form a single circumscribing circular boundary (but with two small gaps).

To give an idea of the broad class of two-pore shapes described by formula (3.1), figure 3 shows a superposition of possible pore shapes for the values *ρ*=0.005, 0.05, 0.1 and 0.2 and for the two choices *θ*=*π*/3 and *θ*=2*π*/3. For the latter choice of *θ* the transition, as *ρ* increases from zero, from small elliptical pores (having semi-axes in the ratio 1 : 3) to near-touching pores exhibiting several high curvature boundary portions is clear. Figure 4 shows the pore configuration for *ρ*=0.5 and *θ*=2*π*/3. An interesting feature is that the two ‘outer’ boundaries of the two pores, when considered together, appear to make up an *almost* closed circumscribing curve that is very close to circular. As *ρ* increases further, the three near-cusps on each pore boundary draw closer together until they almost touch.

It should be emphasized that, while we focus here on the special choice (3.6), generalizing the following analysis to any other value of *θ* is completely straightforward.

The special family of shapes we have just chosen affords us an opportunity to study stress interaction effects between two highly non-circular pores. Remarkably, those stress fields can be found in explicit analytical form for the two physically interesting scenarios examined in §§5 and 6.

## 4. Complex potentials in plane elasticity

The stresses and displacements under plane strain or plane stress conditions of a two-dimensional material can be expressed in terms of two complex potentials *ϕ*(*z*) and *ψ*(*z*). The displacements are given by

4.1 $$2G(u+iv)=\kappa \phi (z)-z\bar{\phi^{\prime }(z)}-\bar{\psi (z)},$$

where the displacement components are denoted by (*u*,*v*), *G* is the shear modulus of the host material, and the so-called Kolosov constant [1] *κ*=3−4*ν* (for plane strain) and *κ*=(3 − *ν*)/(1 + *ν*) (for plane stress), with *ν* being the Poisson ratio. The potentials *ϕ*(*z*) and *ψ*(*z*) can be found by solving boundary value problems of different type involving the surface tractions. We now consider two such problems separately.

## 5. Two uniformly pressurized pores

Consider an infinite elastic body containing two pores, with no stresses acting at infinity, and with a uniform hydrostatic pressure of magnitude *p*=1 acting along the pore boundaries. We will find the stress distribution around the pores subject to this uniform hydrostatic loading. Our results are a generalization, to two non-circular pores, of the work of Ekneligoda & Zimmerman [10] who studied single isolated pores.

In [10], it is shown that for problems in which the traction is specified along the contour then

5.1 $$\phi (z)+z\bar{\phi^{\prime }(z)}+\bar{\psi (z)}\;=\;f_{x}+if_{y}\equiv F,$$

where *F* equals *i* times the integral, starting from some arbitrary boundary point, of the complex traction along the boundary. For hydrostatic pressure of unit magnitude the relevant *F*=−*z* [2,10], so that the boundary condition takes the form

5.2 $$\phi (z)+z\bar{\phi^{\prime }(z)}+\bar{\psi (z)}=-z.$$

For us, there is an important difference to the treatment in [10] arising from the fact that the elastic medium we consider is doubly connected; the analysis of multiply connected geometries is discussed in detail in the standard texts [1,2]. Owing to some additive freedoms in the specification of the potentials *ϕ*(*z*) and *ψ*(*z*), a constant of integration that would ordinarily appear in (5.2) has there been set to zero with impunity. However, for problems with multiple boundaries, only one such constant can be taken to vanish leaving others to be determined.

For the problem of two symmetric pores considered here, it turns out that the relevant conditions on the pore boundaries can be taken to be

5.3 $$\begin{array}{rl} \phi (z)+z\bar{\phi^{\prime }(z)}+\bar{\psi (z)} & =-z+\gamma ,\;\mathrm{on}\;\mathrm{pore}\;1\\ \text{and}\;\;\phi (z)+z\bar{\phi^{\prime }(z)}+\bar{\psi (z)} & =-z-\gamma ,\;\mathrm{on}\;\mathrm{pore}\;2, \end{array}\}$$

where *γ* is some real constant to be found and, clearly, neither of the integration constants have been set equal to zero here (unless it turns out that *γ*=0—the determination of *γ* is discussed below). This is because the symmetry of the geometrical configuration with respect to rotation by *π* around the origin can be used to argue that *ϕ*(*z*) and *ψ*(*z*) can be chosen to be odd functions:

5.4 ϕ(−z)=−ϕ(z),ψ(−z)=−ψ(z)

with simple zeros as z→∞. (5.4) requires that

5.5 ϕ(0)=0,ψ(0)=0

which fixes all additive degrees of freedom in *ϕ*(*z*) and *ψ*(*z*) and necessitates the appearance of ±*γ* in (5.3). The fact that the two constants are negatives of each other follows from (5.4) and the symmetry of the pores.

We now introduce the functions

5.6 Φ(ζ)≡ϕ(z(ζ)),Ψ(ζ)≡ψ(z(ζ)).

Because it is a one-to-one conformal mapping, *z*(*ζ*) is analytic everywhere in the annulus *ρ*<|*ζ*|<1 except for the required simple pole at $\zeta =\sqrt{\rho }$ which maps to z=∞. But because *ϕ*(*z*) and *ψ*(*z*) are analytic in the elastic medium, and decay as z→∞, the composed functions *Φ*(*ζ*) and *Ψ*(*ζ*) turn out to be *analytic* everywhere in the annulus *ρ*<|*ζ*|<1.

In terms of the functions (5.6), the complex conjugate of the first boundary condition in (5.3) takes the form

5.7 $$\bar{\Phi }(1/\zeta )+\bar{z}(1/\zeta )\frac{\Phi^{\prime }(\zeta )}{z^{\prime }(\zeta )}+\Psi (\zeta )=-\bar{z}(1/\zeta )+\bar{\gamma },\;\mathrm{on}\;C_{0},$$

where we have used the fact that $\bar{\zeta }=1/\zeta$ on *C*_0_. The complex conjugate of the second boundary condition in (5.3) becomes

5.8 $$\bar{\Phi }(\rho^{2}/\zeta )+\bar{z}(\rho^{2}/\zeta )\frac{\Phi^{\prime }(\zeta )}{z^{\prime }(\zeta )}+\Psi (\zeta )=-\bar{z}(\rho^{2}/\zeta )-\bar{\gamma },\;\mathrm{on}\;C_{1},$$

where we have used the fact that $\bar{\zeta }=\rho^{2}/\zeta$ on *C*_1_. On use of property (3.5) of the conformal mapping function, (5.8) becomes

5.9 $$\bar{\Phi }(\rho^{2}/\zeta )+\bar{z}(1/\zeta )\frac{\Phi^{\prime }(\zeta )}{z^{\prime }(\zeta )}+\Psi (\zeta )=-\bar{z}(1/\zeta )-\bar{\gamma }.$$

Subtraction of (5.7) and (5.9) now leads to

5.10 $$\bar{\Phi }(\rho^{2}/\zeta )-\bar{\Phi }(1/\zeta )=-2\bar{\gamma }\;\mathrm{or}\;\Phi (\rho^{2}\zeta )=\Phi (\zeta )-2\gamma .$$

This is a key observation that underlies the success of our approach. It should be clear from the sequence of steps above that (5.10) is a consequence of the condition (3.5) pertaining to the class of quadrature domains to which we have restricted attention.

As a point of interest, meromorphic functions satisfying the second of the functional relations in (5.10)—which reduces to (2.9) when *γ*=0—are sometimes called *quasi-loxodromic* functions.

In view of (5.10), the analyticity properties of *Φ*(*ζ*) throughout the complex plane will be determined by its analyticity properties in the annulus *ρ*<|*ζ*|<1/*ρ*. From the complex conjugate of (5.7), we find

5.11 $$\Phi (\zeta )=-z(\zeta )-z(\zeta )\frac{{\bar{\Phi }}^{\prime }(1/\zeta )}{{\bar{z}}^{\prime }(1/\zeta )}-\bar{\Psi }(1/\zeta )+\gamma .$$

But, *Φ*(*ζ*) and *Ψ*(*ζ*) are both known to be analytic in *ρ*<|*ζ*|<1, so that

5.12 $$\frac{{\bar{\Phi }}^{\prime }(1/\zeta )}{{\bar{z}}^{\prime }(1/\zeta )},\;\bar{\Psi }(1/\zeta )$$

are analytic in the annulus 1<|*ζ*|<1/*ρ* (recall also that, for a one-to-one conformal mapping, *z*′(*ζ*)≠0 for *ρ*<|*ζ*|<1). Hence, in the annulus 1<|*ζ*|<1/*ρ*, (5.11) implies that the only possible singularities of *Φ*(*ζ*) are those inherited from *z*(*ζ*). However, (3.1) reveals that *z*(*ζ*) has just two simple pole singularities there at the points $e^{\pm i\theta }/\sqrt{\rho }$. It is then natural to propose that

5.13 $$\Phi (\zeta )=AK(\zeta \sqrt{\rho }\;e^{i\theta })+BK(\zeta \sqrt{\rho }\;e^{-i\theta })+C,$$

for some constants *A*,*B* and *C* and where *K* is the function defined in (2.3). This form has the required simple poles at $e^{\pm i\theta }/\sqrt{\rho }$ forced there by the presence of the two *K*-functions. On use of the first of the properties (2.5) of *K*(*ζ*), *Φ*(*ζ*) satisfies (5.10) provided that

5.14 −A−B=−2γ.

*A* and *B* can be found by equating the residues of the simple poles at $e^{\pm i\theta }/\sqrt{\rho }$ on each side of equation (5.11). Indeed, it is found that

5.15 $$A=-{\left[\frac{L(\rho )+L(\rho \;e^{-2i\theta })}{X}+\frac{e^{i\theta }}{\sqrt{\rho }\bar{a}}\right]}^{-1},\;B=A,\;\gamma =A,$$

where *a* is such that near $\zeta =e^{-i\theta }/\sqrt{\rho }$,

5.16 $$z(\zeta )∼\frac{a}{\zeta -e^{-i\theta }/\sqrt{\rho }}+\mathrm{analytic}\;\mathrm{function},$$

(a formula for *a* is found in appendix B) and where $X=\sqrt{\rho }\;e^{-i\theta }z^{\prime }(\sqrt{\rho }\;e^{-i\theta })$ or, explicitly,

5.17 $$\begin{array}{rl} X & =\frac{RP(-e^{i\theta })P(\rho \;e^{i\theta })P(-\rho \;e^{i\theta })}{P(e^{i\theta })P(\rho \;e^{2i\theta })P(\rho )}[K(-e^{i\theta })+K(\rho \;e^{i\theta })+K(-\rho \;e^{i\theta })-K(e^{i\theta })-K(\rho \;e^{2i\theta })-K(\rho )], \end{array}$$

where we have used the fact that

5.18 $$\begin{array}{rl} \zeta z^{\prime }(\zeta ) & =z(\zeta )[K(-\zeta /\sqrt{\rho })+K(\zeta \sqrt{\rho })+K(-\zeta \sqrt{\rho })-K(\zeta /\sqrt{\rho })-K(\zeta \sqrt{\rho }\;e^{i\theta })-K(\zeta \sqrt{\rho }\;e^{-i\theta })]. \end{array}$$

The constant *C* follows from ensuring that *ϕ*(0)=0 as required in (5.5) is satisfied. Because $\zeta =-\sqrt{\rho }$ is the pre-image of *z*=0, then

5.19 $$C=-A[K(-\rho \;e^{i\theta })+K(-\rho \;e^{-i\theta })].$$

Finally, with *Φ*(*ζ*) given by (5.13), *Ψ*(*ζ*) follows from (5.7):

5.20 $$\Psi (\zeta )=-\bar{z}(1/\zeta )-\bar{\Phi }(1/\zeta )-\bar{z}(1/\zeta )\frac{\Phi^{\prime }(\zeta )}{z^{\prime }(\zeta )}+A.$$

In summary, we have found that the complex potentials for two pores under hydrostatic loading are

5.21 $$\begin{array}{rl} \phi (z(\zeta )) & =\Phi (\zeta )=A[K(\zeta \sqrt{\rho }\;e^{i\theta })+K(\zeta \sqrt{\rho }\;e^{-i\theta })-K(-\rho \;e^{i\theta })-K(-\rho \;e^{-i\theta })]\\ \text{and}\;\;\psi (z(\zeta )) & =\Psi (\zeta )=-\bar{z}(1/\zeta )-\bar{\Phi }(1/\zeta )-\bar{z}(1/\zeta )\frac{\Phi^{\prime }(\zeta )}{z^{\prime }(\zeta )}+A, \end{array}\}$$

where *A* is given in (5.15).

With the stress fields determined in this way, it is possible to compute the pore compressibility *C*_pc_ [10] defined by

5.22 $$C_{\mathrm{pc}}=C_{pp}+\frac{1-2\nu }{G},$$

where

5.23 Cpp=ΔAA≡1A∮pore 1u.n ds=1ARe[∮|ζ|=1(u−iv)iz′(ζ) dζ],

and

5.24 $$A=\frac{1}{2i}\oint_{\mathrm{pore}\;1}\bar{z}\;dz=-\frac{1}{2i}\oint_{|\zeta |=1}\bar{z}z^{\prime }(\zeta )\;d\zeta ,$$

is the initial area of pore 1 in figure 1 (taken to be the image of |*ζ*|=1). These quantities were found in [10] for the class of isolated pores studied there. For plane strain, it is preferable to consider the non-dimensionalized quantity

5.25 $$\frac{GC_{\mathrm{pc}}}{1-\nu }$$

because this turns out to be purely a function of the pore geometry. Zimmerman [9] showed this in the single pore case using algebraic manipulations based on a conformal mapping representation, but it pertains to the multiple pore case too as we show in appendix C using more general arguments. Appendix C gives details of how, on substituting for *Ψ*(*ζ*) from (5.20) and use of the fact that *κ*+1=4(1−*ν*) for plane strain, to establish that

5.26 Cpc=ΔAA+1−2νG=(1−ν)G[2AIm[∮pore 1Φ¯ dz]+2]

implying that the quantity of interest (5.25) is

5.27 GCpc1−ν=2+2AIm[∮pore 1Φ¯ dz].

The right-hand side of (5.27) is purely a function of the pore geometry and is independent of the Poisson ratio.

To compute the values of the right-hand side of (5.27), whose integrand is known explicitly in terms of the special functions *P*(*ζ*),*K*(*ζ*) and *L*(*ζ*), we can make use of convenient infinite sum representations of *K*(*ζ*) and *L*(*ζ*) are given in (.9) of appendix A. Truncated versions of these can be used for numerical computation. *P*(*ζ*) can similarly be evaluated easily by truncating the infinite product (2.1) that defines it. The explicit integral in (5.27) can be evaluated, to exponential accuracy, by the trapezoidal rule.

**Figure 5.**
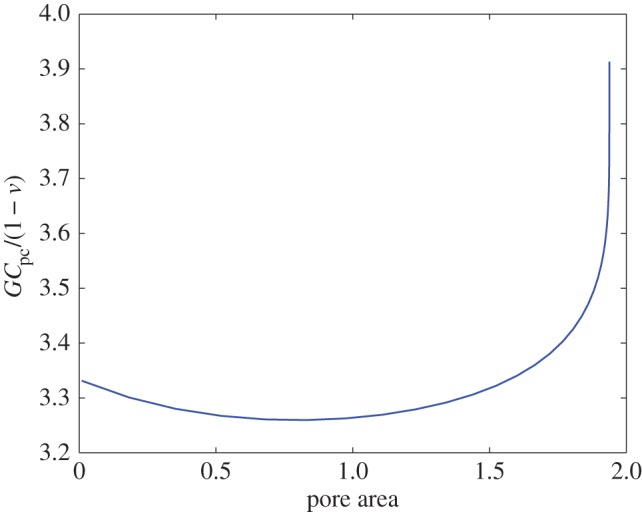
Graph of the normalized compressibility *GC*_pc_/(1−*ν*) against the area A of a single pore for *θ*=2*π*/3.

 Figure 5 shows a graph of *GC*_pc_/(1−*ν*), computed using (5.27), against pore area A for *θ*=2*π*/3. Here, *ρ* is used as a parameter: for each *ρ*, the value of *R* is chosen that fixes the centroids of the pores to be at ±1. The centroid position C of pore 1 is readily computed from the formula

5.28 $$C=\frac{\oint_{\mathrm{pore}\;1}z\bar{z}\;dz}{\oint_{\mathrm{pore}\;1}\bar{z}\;dz}=\frac{\oint_{|\zeta |=1}z(\zeta )z(1/\zeta )z^{\prime }(\zeta )\;d\zeta }{\oint_{|\zeta |=1}z(1/\zeta )z^{\prime }(\zeta )\;d\zeta },$$

where we have used (3.4). The parameter *R* can be chosen, as an explicit function of *ρ* and *θ*, so that C=1 for pore 1. The conformal map (3.1) is then fully determined and the pore area A can be found from (5.24).

As *ρ*→0, so that the pore area tends to zero, the shapes of the pores to which we have restricted attention tend to ellipses elongated in the *y*-direction with major axis three times larger than the minor axis; this can be seen by simply inspecting plots of the images under the mapping (3.1), or by analysis of this formula as *ρ*→0. This feature can be seen from figure 3 where the small-area pores appear to be elliptical in shape. In this limit, the graph tends to the value 10/3 in agreement with the results of Zimmerman [9] for an isolated ellipse under a hydrostatic loading. He showed that for the conformal mapping to the exterior of an ellipse of the form

5.29 $$z=h(\zeta )=\frac{1}{\zeta }+a_{1}\zeta$$

then

5.30 $$\frac{GC_{\mathrm{pc}}}{1-\nu }=2\left(\frac{1+a_{1}^{2}}{1-a_{1}^{2}}\right)$$

and, for an ellipse with semi-axes in the ratio 1 to 3, then $a_{1}=-\frac{1}{2}$. This feature, which serves as a check on our more general mathematical approach, is to be expected, because in this small area limit, the typical lengthscale of each pore is small compared with their separation meaning that they become increasingly unaware of each other's presence. Then, each pore acts like an isolated pore under uniform hydrostatic pressure.

As the pore area increases from zero the interaction effects and shape deformations lead to a net decrease in compressibility. However, eventually, as the pore areas increase further the interaction effects lead to an increase of the compressibility above the starting value of 10/3 relevant for small pore areas. The limit *ρ*→1^−^ is a singular mathematical limit and must be treated with care; the very steep vertical slope of the graph in figure 5 is indicative of this. In particular, it should be noted that for the validity of our two-pore analysis, we require *ρ* to be strictly less than unity on topological grounds; moreover, the rate of convergence of the infinite product (2.2) deteriorates as *ρ*→1^−^. In all calculations used to plot figure 5, we allowed *ρ* to increase as high as 0.9 and it is interesting that, as *ρ* becomes larger, the two pores almost merge in such a way that the circumscribed boundary tends to that of a single *circular* pore. Figure 5 shows that the associated compressibility appears to tend to the value 4 and that behaviour might also be expected: while the compressibility of a purely circular pore is well known to be 2 [9,10] the apparent factor-of-2 discrepancy arises only, because we have normalized the pore compressibility with respect to the area A of a *single pore*. However, it is clear from the geometry of figure 4 that this is precisely *half* the area of the full circumscribed semicircle marked out by the ‘exterior part’ of each pore boundary.

In both limits *ρ*→0 and *ρ*→1^−^, the analytical solutions for two pores therefore agree with the relevant single pore results lending us confidence in the analysis. The compressibility of other classes of pore shapes can be studied using a similar analysis. This is discussed further in §7.

## 6. Two pores subject to far-field shear

As a second example of the versatility of our construction, consider the same class of elastic bodies with two pores but now with an imposed state of pure shear stress at infinity. The following results generalize, to two non-circular pores, the work of Ekneligoda & Zimmerman [11].

Following Ekneligoda & Zimmerman [11], we consider two problems, called problems 1 and 2. In problem 1, the far-field shear is taken to be aligned with the real and imaginary axes, so that *τ*_*xy*_=*τ*_*yx*_=*τ* and the relevant complex potentials are simply

6.1 $$\tilde{\phi }(z)=0,\;\tilde{\psi }(z)=i\tau z.$$

The stress state of problem 1 has the required form at infinity, but produces an unwanted traction on the boundaries of the two pores. Problem 2, which is now defined in the elastic medium containing the two pores, is taken to have zero stress at infinity and has boundary tractions that, when added to those of problem 1, cancel them out. By linearity, the solution for a body with two traction-free pores and the required shear stress at infinity is given by the sum of the solutions to problems 1 and 2.

The potentials $\tilde{\phi }(z)$ and $\tilde{\psi }(z)$ in (6.1) solve problem 1, but it remains to solve problem 2, whose potentials will be denoted by *ϕ*(*z*) and *ψ*(*z*). In this situation, the boundary conditions on the two pores boundaries are now

6.2 $$\begin{array}{rl} \phi (z)+z\bar{\phi^{\prime }(z)}+\bar{\psi (z)} & =i\tau \bar{z}+\gamma ,\;\mathrm{onpore}\;1\\ \text{and}\;\;\phi (z)+z\bar{\phi^{\prime }(z)}+\bar{\psi (z)} & =i\tau \bar{z}-\gamma ,\;\mathrm{onpore}\;2. \end{array}\}$$

Again, we can argue on the basis of symmetry that *ϕ*(*z*) and *ψ*(*z*) are odd, and the constants in (6.2) are chosen to be consistent with this symmetry.

On introduction of the functions defined in (5.6), the complex conjugate of the first boundary condition in (6.2) is

6.3 $$\bar{\Phi }(1/\zeta )+\bar{z}(1/\zeta )\frac{\Phi^{\prime }(\zeta )}{z^{\prime }(\zeta )}+\Psi (\zeta )=-i\tau z(\zeta )+\bar{\gamma },\;\mathrm{on}\;C_{0},$$

where we have used the fact that $\bar{\zeta }=1/\zeta$ on *C*_0_. The complex conjugate of the second boundary condition in (6.2) is

6.4 $$\bar{\Phi }(\rho^{2}/\zeta )+\bar{z}(\rho^{2}/\zeta )\frac{\Phi^{\prime }(\zeta )}{z^{\prime }(\zeta )}+\Psi (\zeta )=-i\tau z(\zeta )-\bar{\gamma },\;\mathrm{on}\;C_{1},$$

where we have used the fact that $\bar{\zeta }=\rho^{2}/\zeta$ on *C*_1_.

On use of the key property (3.5) of the conformal mapping function, (6.4) becomes

6.5 $$\bar{\Phi }(\rho^{2}/\zeta )+\bar{z}(1/\zeta )\frac{\Phi^{\prime }(\zeta )}{z^{\prime }(\zeta )}+\Psi (\zeta )=-i\tau z(\zeta )-\bar{\gamma }.$$

Hence, (6.3) and (6.5) again lead to the result

6.6 $$\Phi (\rho^{2}\zeta )=\Phi (\zeta )-2\gamma .$$

The complex conjugate of (6.3) implies that

6.7 $$\Phi (\zeta )=-z(\zeta )\frac{{\bar{\Phi }}^{\prime }(1/\zeta )}{{\bar{z}}^{\prime }(1/\zeta )}-\bar{\Psi }(1/\zeta )+i\tau \bar{z}(1/\zeta )+\gamma .$$

By inspection, we see that *Φ*(*ζ*) now inherits the singularity structure of *both* *z*(*ζ*) and $\bar{z}(1/\zeta )$ in the annulus 1<|*ζ*|<1/*ρ*: it, therefore, has *three* simple poles located at $1/\sqrt{\rho },e^{\pm i\theta }/\sqrt{\rho }$. It is natural to pose that

6.8 $$\Phi (\zeta )=AK(\zeta \sqrt{\rho })+BK(\zeta \sqrt{\rho }e^{i\theta })+CK(\zeta \sqrt{\rho }\;e^{-i\theta })+D$$

for some complex constants *A*,*B*,*C* and *D* that satisfy

6.9 −A−B−C=−2γ.

The appearance of the three *K*-functions means that *Φ*(*ζ*) has the required simple pole singularities; the constraint (6.9) derives from the requirement that *Φ*(*ζ*) satisfies (6.6). The constants *A*,*B* and *C* are determined by equating residues of the left- and right-hand sides of (6.7) at the three poles at $1/\sqrt{\rho },e^{\pm i\theta }/\sqrt{\rho }$. After some straightforward algebra to compute residues, it is found that

6.10 $$A=i\tau \sqrt{\rho }b,\;B=\frac{i\tau \rho ab[\bar{a}\sqrt{\rho }(L(\rho )-L(\rho \;e^{-2i\theta }))+e^{i\theta }X]}{|X-\bar{a}\sqrt{\rho }\;e^{-i\theta }L(\rho \;e^{-2i\theta }){|}^{2}-|a{|}^{2}\rho L{\left(\rho \right)}^{2}},\;C=-\bar{B},$$

where *b* is such that near $\zeta =1/\sqrt{\rho }$ we have

6.11 $$\bar{z}(1/\zeta )∼\frac{b}{\zeta -1/\sqrt{\rho }}+\text{analytic function},$$

(a formula for *b* is given in appendix B) and *X* is the same quantity defined in (5.17). From (6.9), we find

6.12 $$\gamma =\frac{A}{2}+\frac{B-\bar{B}}{2}$$

which is purely imaginary.

With *A*,*B* and *C* determined in (6.10), *D* is determined from (5.5):

6.13 $$\Phi (-\sqrt{\rho })=AK(-\rho )+BK(-\rho \;e^{i\theta })+CK(-\rho \;e^{-i\theta })+D=0.$$

Finally, with both *Φ*(*ζ*) and *z*(*ζ*) now known explicitly, *Ψ*(*ζ*) is given by (6.3):

6.14 $$\Psi (\zeta )=-i\tau z(\zeta )-\gamma -\bar{\Phi }(1/\zeta )-\bar{z}(1/\zeta )\frac{\Phi^{\prime }(\zeta )}{z^{\prime }(\zeta )}.$$

In summary, we have found the stress fields for Problem 2 associated with two pores with an imposed state of pure shear at infinity to involve the two complex potentials

6.15 $$\begin{array}{rl} \Phi (\zeta ) & =A(K(\zeta \sqrt{\rho })-K(-\rho ))+B(K(\zeta \sqrt{\rho }\;e^{i\theta })-K(-\rho \;e^{i\theta }))-\bar{B}(K(\zeta \sqrt{\rho }\;e^{-i\theta })-K(-\rho \;e^{-i\theta }))\\ \text{and}\;\;\Psi (\zeta ) & =-i\tau z(\zeta )-\bar{\Phi }(1/\zeta )-\bar{z}(1/\zeta )\frac{\Phi^{\prime }(\zeta )}{z^{\prime }(\zeta )}-\frac{A}{2}-\frac{B-\bar{B}}{2}, \end{array}\}$$

with *A* and *B* given explicitly in (6.10).

With the stress fields determined explicitly in this way, the shear compliance of the various pore configurations can be studied in the spirit of the single pore analysis of Ekneligoda & Zimmerman [11].

## 7. Quadrature domains

Where did formula (3.1) come from? And how can other formulae, describing other pore shapes, be derived for which the analysis of this paper also works?

To answer these questions, we remark that the foregoing analysis has implicitly demonstrated the role played, in problems of linear plane elasticity, by a special class of doubly connected planar domains produced as the images of conformal mappings of the loxodromic form (3.1). This family of elastic bodies is just one example of a much broader class of planar domains known as (multiply connected) *quadrature domains* [18,20]. Other quadrature domains will similarly give rise, in principle, to solutions for the elastic stress problems considered here that are expressible in closed form. The question of how to *construct* multiply connected quadrature domains has been a topic of much recent research interest [18,20]. That constructive theory led to formula (3.1).

To explain this in more detail, note that we have focused here on the example of two pores as this case affords the clearest insights into the associated function theory. But, all the same mathematical ideas pertain to the case of *any* finite distribution of pores. If *M*+1 pores are present, with *M*>1, then to generalize our analysis the surrounding elastic medium should be taken to be an unbounded (*M*+1)-connected quadrature domain. Then, for the conformal mapping, a suitable choice of pre-image *ζ* domain is the unit *ζ*-disc now with *M* smaller circular discs excised [23,24]; the annulus *ρ*<|*ζ*|<1 used here is the unit disc with just a single, concentric, circular disc excised. It is possible to associate, to *any* such circular pre-image domain, a function called the Schottky–Klein prime function [23], often denoted as a function of just two variables, *ω*(*ζ*,*α*), even though it also depends on the choice of the circular pre-image domain (i.e. the geometry of the pre-image circles). To within a constant of proportionality, the function *P*(*ζ*/*α*), which has clearly played a crucial role in our analysis, is exactly the Schottky–Klein prime function [23] associated with the concentric annulus *ρ*<|*ζ*|<1. Crowdy & Marshall [24] have given a detailed account of how to construct conformal mappings to multiply connected quadrature domains using the Schottky–Klein prime function as a building block. Those conformal mappings have the property of being *automorphic functions*; in the two-pore case, those automorphic functions are precisely the loxodromic functions considered here.

Indeed, Crowdy & Marshall [25] have shown that the very same class of mappings (3.1) explored here for these plane elasticity problems also provide analytical solutions of the two-dimensional Euler equations of fluid dynamics describing two rotating vortex patches. They also showed how that work can be extended to the case of *any number* of co-rotating vortex patches in equilibrium [26]; to do so, use is made of multiply connected quadrature domains and the function theory based on the Schottky–Klein prime function just described [24,23]. In the same way, the mathematics of Crowdy & Marshall [26] can be exploited to solve plane elasticity problems for elastic bodies with multiple pores by extension of the methodology of the present paper. This is left as the topic for future work.

It is important to point out a significant mathematical fact: Gustafsson [27] has shown that the class of multiply connected quadrature domains is dense (in an appropriate sense [27]) in the class of all multiply connected planar domains (with sufficiently smooth boundary components). To get a flavour of this in the doubly connected case, we note that generalizations of (3.1) that are also loxodromic functions (and, therefore, correspond to doubly connected quadrature domains) are given by (see [24] for more details):

7.1 $$z=z(\zeta )=R\left[\frac{P(\zeta /\alpha )}{P(\zeta /\beta )}\right]\prod_{k=1}^{N}\frac{P(\zeta /s_{k})}{P(\zeta /r_{k})},$$

where *α* maps to *z*=0, whereas *β* maps to z=∞ (with *ρ*<|*α*|,|*β*|<1) and, in order that the function is loxodromic, the other poles {*r*_*k*_|*k*=1,…,*N*} and zeros {*s*_*k*_|*k*=1,…,*N*} of the mapping (7.1) must be in the annulus 1<|*ζ*|<1/*ρ* and satisfy the single condition

7.2 $$\alpha \prod_{k=1}^{N}s_{k}=\beta \prod_{k=1}^{N}r_{k}.$$

By taking *N* sufficiently large, and by appropriate choices of the parameters *R*,*ρ*,*α*,*β*, {*r*_*k*_,*s*_*k*_|*k*=1,…,*N*}, approximate conformal mappings of this type to any unbounded doubly connected region can be found, in principle. Then, the analysis of this paper can be repeated for the new mappings (7.1) to find, also in terms of the Schottky–Klein prime function, analytical expressions for the complex potentials for the stress fields.

In this way, it is possible, in principle at least, to find approximating analytical solutions to the stress problems of plane elasticity in *any* multiply connected region by identifying a satisfactory quadrature domain approximant and generalizing the solution scheme laid out here. Of course, as quadrature domains with greater numbers of poles are needed to approximate the domains this becomes less feasible in practice. Nevertheless, a broad class of new analytical solutions to the basic problems of plane elasticity is essentially available. Certainly, elastic bodies corresponding to *low order* quadrature domains—that is, those with associated conformal mappings which are meromorphic functions with a small number of poles—can be studied explicitly, as we have done here.

## 8. Discussion

The stress fields around a family of two-pore configurations in an elastic body have been found in analytical form by invoking some novel mathematical ideas from quadrature domain theory.

One parameter in the description of the shapes studied here is the so-called conformal modulus *ρ* [23], which governs the degree of ‘interaction’ of the two pores. Our class of solutions, and its various generalizations, affords an opportunity to study, in an explicit way, how pore interactions affect quantities of physical interest such as compressibility, shear compliance and other measures of excess strain in a body owing to the presence of a pair of cavities. For illustration, we have investigated in detail the compressibility of a particular family of two pore configurations.

An alternative approach to the two-pore problem considered here is via purely numerical schemes, such as boundary integral methods. However, such methods would have difficulty in accurately resolving the stress fields in the vicinity of the high curvature, close-to-touching regions exhibited by the family of pore shapes studied here. Our analytical solutions should serve as valuable benchmarks for any such numerical formulations.

Finally, it is not without import to remark that the mathematical approach expounded here also works, of course, for *simply connected* quadrature domains. It is a basic fact [28,18] that the boundaries of simply connected quadrature domains can be parametrized by rational function conformal mappings. The function theory in that case becomes significantly easier with the solutions for *Φ*(*ζ*) and *Ψ*(*ζ*) also being rational functions. But then the approach we have demonstrated here reduces to ideas that are already well known in solid mechanics; see, for example, the review on the bending of thin plates using the so-called ‘rational function method’ by Hasebe & Wang [29]. What we have shown here is that if one wants to extend the rational function method to *multiply connected* regions, then the natural mathematical generalization is to consider the class of multiply connected quadrature domains; the boundaries of those domains can be parametrized by so-called automorphic functions [,^23^,^24^,^26^] (i.e. the loxodromic functions of this paper) which are the natural generalizations of rational functions to higher-genus Riemann surfaces. The ideas of this paper therefore form the basis of what one might call the ‘loxodromic function method’ for doubly connected regions or the ‘automorphic function method’ for higher connected regions approximated by multiply connected quadrature domains.

## Data accessibility

This article contains no external data. Analytical solutions are reported, and all graphs can be directly reproduced by calculation based on the explicit formulae.

## Appendix A. Properties of *P*(*ζ*)

For completeness, we show how to derive equations (2.2), (2.5) and (2.7). The definition (2.1) of *P*(*ζ*) implies

A 1 $$P(\rho^{2}\zeta )=(1-\rho^{2}\zeta )\prod_{k=1}^{\infty }(1-\rho^{2k}\rho^{2}\zeta )(1-\rho^{2k}\rho^{-2}\zeta^{-1}).$$

On use of the identities

A 2 $$\begin{array}{rl} \prod_{k=1}^{\infty }(1-\rho^{2k}\rho^{-2}\zeta^{-1}) & =(1-\zeta^{-1})\prod_{k=1}^{\infty }(1-\rho^{2k}\zeta^{-1})\\ \text{and}\;\;(1-\rho^{2}\zeta )\prod_{k=1}^{\infty }(1-\rho^{2k}\rho^{2}\zeta ) & =\prod_{k=1}^{\infty }(1-\rho^{2k}\zeta ), \end{array}\}$$

it can be shown that the right-hand side of (A 1) becomes

A 3 $$\left(1-\zeta^{-1})\prod_{k=1}^{\infty }(1-\rho^{2k}\zeta^{-1})(1-\rho^{2k}\zeta \right)$$

and the first identity of (2.2) follows.

The second identity in (2.2) is a direct consequence of the following invariance:

A 4 $$\frac{P(\zeta )}{1-\zeta }=\frac{P(\zeta^{-1})}{1-\zeta^{-1}}.$$

Differentiation of the first of the identities (2.2) with respect to *ζ* yields

A 5 $$\rho^{2}P^{\prime }(\rho^{2}\zeta )=\zeta^{-2}P(\zeta )-\zeta^{-1}P^{\prime }(\zeta )$$

which, on division by the equation

A 6 $$\zeta^{-1}P(\rho^{2}\zeta )=-\zeta^{-2}P(\zeta ),$$

leads to the first of the identities in (2.5). Differentiation of the second of the identities (2.2) with respect to *ζ*, we find

A 7 $$-\zeta^{-2}P^{\prime }(\zeta^{-1})=\zeta^{-2}P(\zeta )-\zeta^{-1}P^{\prime }(\zeta )$$

which, on division by the equation

A 8 $$-\zeta^{-1}P(\zeta^{-1})=\zeta^{-2}P(\zeta ),$$

yields the second of the identities in (2.5).

Finally, the identities (2.7) follow by differentiating (2.5) with respect to *ζ*.

It can be shown, directly from their definitions and the infinite product (2.2), that convenient infinite sum expressions for *K* and *L* are

A 9 $$\begin{array}{rl} K(\zeta ) & =-\frac{\zeta }{1-\zeta }+\sum_{n=1}^{\infty }\left(-\frac{\rho^{2n}\zeta }{1-\rho^{2n}\zeta }+\frac{\rho^{2n}/\zeta }{1-\rho^{2n}/\zeta }\right)\\ \text{and}\;\;L(\zeta ) & =-\sum_{n=0}^{\infty }\frac{\rho^{2n}\zeta }{{\left(1-\rho^{2n}\zeta \right)}^{2}}-\sum_{n=1}^{\infty }\frac{\rho^{2n}/\zeta }{{\left(1-\rho^{2n}/\zeta \right)}^{2}}. \end{array}\}$$

As a final remark, by employing appropriate transformations of the parameters and of the independent variable, the functions *P*(*ζ*),*K*(*ζ*) and *L*(*ζ*) can be identified with the Weierstrass-*σ*, Weierstrass-*ζ* and Weierstrass-P functions, respectively [22,23].

## Appendix B. Formulae for *a* and *b*

Equation (3.1) implies that we can write

B 1 $$z(\zeta )=R\left[\frac{P(-\zeta /\sqrt{\rho })P(-\zeta \sqrt{\rho })P(\zeta \sqrt{\rho })}{\hat{(}1-\zeta \sqrt{\rho }\;e^{i\theta })\hat{P}(\zeta \sqrt{\rho }\;e^{i\theta })P(\zeta /\sqrt{\rho })P(\zeta \sqrt{\rho }\;e^{-i\theta })}\right].$$

Hence, near $\zeta =e^{-i\theta }/\sqrt{\rho }$,

B 2 $$z∼\frac{a}{\zeta -e^{-i\theta }/\sqrt{\rho }},$$

where

B 3 $$a=-\frac{R}{\sqrt{\rho }\;e^{i\theta }}\left[\frac{P(-e^{-i\theta }/\rho )P(e^{-i\theta })P(-e^{-i\theta })}{\hat{P}(1)P(e^{-i\theta }/\rho )P(e^{-2i\theta })}\right].$$

Similarly, near $\zeta =e^{i\theta }/\sqrt{\rho }$,

B 4 $$z∼\frac{\bar{a}}{\zeta -e^{i\theta }/\sqrt{\rho }}.$$

Similarly, (3.1) implies that

B 5 $$\bar{z}(1/\zeta )=R\left[\frac{P(-1/[\zeta \sqrt{\rho }])P(-\sqrt{\rho }/\zeta )P(\sqrt{\rho }/\zeta )}{\left(1-1/[\zeta \sqrt{\rho }])\hat{P}(1/[\zeta \sqrt{\rho }])P(\sqrt{\rho }\;e^{i\theta }/\zeta )P(\sqrt{\rho }\;e^{-i\theta }/\zeta \right)}\right]$$

or

B 6 $$\bar{z}(1/\zeta )=R\left[\frac{\zeta P(-1/[\zeta \sqrt{\rho }])P(-\sqrt{\rho }/\zeta )P(\sqrt{\rho }/\zeta )}{\left(\zeta -1/\sqrt{\rho })\hat{P}(1/[\zeta \sqrt{\rho }])P(\sqrt{\rho }\;e^{i\theta }/\zeta )P(\sqrt{\rho }\;e^{-i\theta }/\zeta \right)}\right].$$

Hence, near $\zeta =1/\sqrt{\rho ,}$ we have

B 7 $$\bar{z}(1/\zeta )∼\frac{b}{\zeta -1/\sqrt{\rho }},$$

where

B 8 $$b=R\left[\frac{P(-1)P(-\rho )P(\rho )}{\sqrt{\rho }\hat{P}(1)P(\rho \;e^{i\theta })P(\rho \;e^{-i\theta })}\right].$$

## Appendix C. Expression for pore compressibility *C*_pc_

Consider a situation in which an elastic medium contains *M*≥1 pores each under uniform hydrostatic pressure of unit strength. Let the boundary of the *j*th pore be ∂*D*_*j*_ and let its area be $A_{j}$. Then, on ∂*D*_*j*_, we have

C 1 $$\phi (z)+z\bar{\phi^{\prime }(z)}+\bar{\psi (z)}=-z+\gamma_{j},\;\mathrm{on}\;\partial D_{j}$$

for some set of constants {*γ*_*j*_|*j*=1,…,*M*}. By definition [10], the compressibility $C_{pp}^{\left(j\right)}$ of the *j*th pore is

C 2 Cpp(j)=1Aj∮∂Dju.n ds=1AjRe[∮∂Dj(u−iv)(−i dz)]

and

C 3 $$C_{\mathrm{pc}}^{\left(j\right)}\equiv C_{pp}^{\left(j\right)}+\frac{1-2\nu }{G},$$

where

C 4 $$u-iv=\frac{1}{2G}[\kappa \bar{\phi (z)}-\bar{z}\phi^{\prime }(z)-\psi (z)],\;A_{j}=\frac{1}{2i}\oint_{\partial D_{j}}\bar{z}\;dz.$$

Hence,

C 5 Cpc(j)=1Aj{Re[∮∂Dj12G[κϕ(z)¯−z¯ϕ′(z)−ψ(z)](−i dz)]+(1−2ν)G12i∮∂Djz¯ dz}.

On substituting for $-\bar{z}\phi^{\prime }(z)-\psi (z)$ from (C 1), it follows that

C 6 Cpc(j)=1Aj{Re[∮∂Dj12G[(κ+1)ϕ(z)¯+z¯−γ¯j](−i dz)]+(1−2ν)G12i∮∂Djz¯ dz}=1Aj{Re[∮∂Dj(κ+1)2Gϕ(z)¯(−i dz)]+(2−2ν)G12i∮∂Djz¯ dz}.

For plane strain *κ*=3−4*ν*, so that

C 7 $$\frac{\kappa +1}{2G}=\frac{2(1-\nu )}{G}$$

and

C 8 Cpc(j)=(1−ν)G{2AjIm[∮∂Djϕ(z)¯ dz]+2}.
